# A Pragmatic Strategy for Improving Diagnosis of Invasive Candidiasis in UK and Ireland ICUs

**DOI:** 10.3390/jof11110784

**Published:** 2025-10-31

**Authors:** Anjaneya Bapat, Timothy W. Felton, Sarah Khorshid, Ignacio Martin-Loeches

**Affiliations:** 1 King’s College Hospital NHS Foundation Trust, London SE5 9RS, UK; anjaneya.bapat@nhs.net; 2 Respiratory Academic Group, Division of Immunology, Immunity to Infection and Respiratory Medicine, University of Manchester, Wythenshawe Hospital, Manchester M23 9LT, UK; tim.felton@manchester.co.uk; 3 Pharmacy Department, St. George’s University Hospitals NHS Foundation Trust, London SW17 0QT, UK; sarah.khorshid@stgeorges.nhs.uk; 4 Department of Intensive Care Medicine, Multidisciplinary Intensive Care Research Organization (MICRO), St. James’s Hospital, D08 NHY1 Dublin, Ireland

**Keywords:** invasive candidiasis, intensive care units, rapid diagnostics, antifungal stewardship, antifungal resistance

## Abstract

Invasive candidiasis (IC) is a life-threatening fungal infection predominantly affecting critically ill patients in intensive care units (ICUs). Despite advances in antifungal therapies, IC remains a diagnostic and therapeutic challenge, with a mortality rate exceeding 40%. The current reliance on blood cultures as the diagnostic gold standard is limited by low sensitivity and prolonged turnaround times, often delaying effective treatment. This often leads to the overuse of empirical antifungal therapies, increasing resistance, healthcare costs, and inconsistent outcomes. To address these issues, this paper introduces a five-step diagnostic strategy developed by an expert panel to optimise IC diagnosis and management. The strategy integrates predictive risk scores, biomarkers, and antifungal susceptibility testing to streamline diagnosis, identify high-risk patients, and promote antifungal stewardship. It also addresses barriers such as resource disparities and variability in clinical practices, offering a practical, standardised strategy for ICUs in the UK and Ireland. The clinical utility of this approach is highlighted through two patient cases. One describes the safe discontinuation of antifungal therapy after a negative (1,3)-β-D-glucan (BDG) assay ruled out IC, reducing unnecessary treatment and adverse effects. The other showcases the use of rapid in-house antifungal susceptibility testing to precisely tailor therapy for a patient with *Nakaseomyces glabratus*, ensuring effective treatment and preventing resistance. This pragmatic five-step guide simplifies and standardises IC diagnosis, aiming to lower mortality, optimise therapies, and promote judicious antifungal use.

## 1. Introduction

Diagnosing invasive candidiasis (IC) remains a significant challenge in intensive care units (ICUs) across the UK and Ireland [1,2,3]. This life-threatening fungal infection, primarily affecting critically ill patients, is the most common critical care-associated fungal disease, with a crude mortality rate ranging from 40% to 55% [1]. Despite advances in antifungal therapies, the incidence of IC continues to rise. Recent data from England highlight an alarming 42% increase in bloodstream infections caused by *Candida* species (spp.) over the past decade, reaching 4.0 cases per 100,000 population in 2022 [4,5,6]. A recent 20-year retrospective population-based study of 2586 *Candida* bloodstream infections revealed shifts in species distribution, with declining *Candida albicans* and *Candida parapsilosis* and rising *Nakaseomyces glabratus* and *Candida dubliniensis* [7]. Mortality remained at 21%, with *C. parapsilosis* linked to lower mortality and *Candida tropicalis* to higher [7].

The limitations of traditional diagnostic methods further complicate early recognition and treatment of IC [6,8]. Blood cultures, though considered the gold standard, suffer from low sensitivity and prolonged turnaround times, making them inadequate for critically ill patients who require rapid intervention. Notably, observational studies have found that the mean time to recovery for *N. glabratus* in blood cultures takes significantly longer than for other *Candida* spp. (e.g., 2.97 ± 1.18 days, compared to 2.17 ± 1.10 days for other *Candida* spp.) [9]. As a result, many clinicians resort to empirical antifungal therapy, which increases the risk of overtreatment, antifungal resistance, and delayed initiation of targeted treatment [10]. In addition to these diagnostic hurdles, there is a lack of standardised protocols across ICUs, leading to inconsistent clinical practices and variability in patient outcomes. To address these challenges, there is an urgent need to develop faster, more accurate diagnostic tools and to equip healthcare professionals with evidence-based strategies for managing IC [8].

In the UK and Ireland, the current diagnostic landscape for IC relies heavily on blood cultures as the gold standard, despite their limitations in sensitivity and turnaround time [6,9]. Non-culture-based tests, such as serum β-D-glucan (BDG) assays, mannan/anti-mannan serological tests, and molecular diagnostics like PCR, are increasingly being used to complement traditional methods [11]. MALDI-TOF MS is also widely adopted for species identification, offering rapid and accurate results [12,13]. These methods, while promising, are not yet uniformly implemented across all ICUs, leading to variability in diagnostic practices and patient outcomes [14].

This paper introduces a pragmatic five-step diagnostic strategy designed specifically for ICUs in the UK and Ireland. By incorporating advanced biomarkers, predictive scoring systems, and a multidisciplinary approach, the strategy aims to streamline the diagnostic process, enhance patient care, and minimise the variability in diagnostic practices. The paper emphasises the importance of integrating innovative diagnostic technologies and implementing antifungal stewardship programmes to reduce the morbidity and mortality associated with IC. Finally, it highlights key knowledge gaps, suggests directions for future research, and advocates for a more structured strategy to IC diagnosis and management.

## 2. Methods

**Figure 1:**
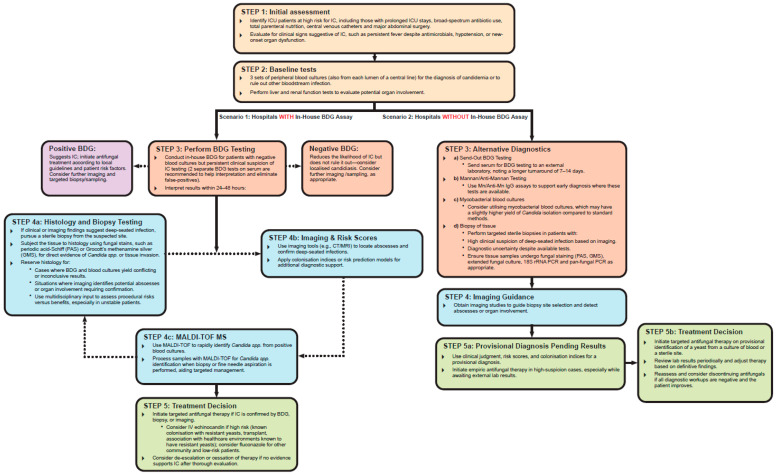
A pragmatic 5-step ‘What to Do’ strategy for diagnosing IC in UK and Ireland ICUs. The time required to make decisions using this strategy depends on the availability of diagnostic tools. For example, BDG assays and MALDI-TOF MS can provide results within 24–48 h in hospitals with in-house capabilities, while external testing may take longer.

A panel of experts from hospitals across the UK and Ireland, specialising in the management of IC in critically ill patients, convened to address the most pressing challenges in diagnosing and treating this condition. Their goal was to develop pragmatic guidance to enhance clinical decision-making and improve patient outcomes. The panel reviewed existing guidelines and critically evaluated their applicability within UK and Irish ICUs. By integrating real-world clinical experience with scientific data, the panel designed a 5-step diagnostic strategy that is both practical and scientifically robust (Figure 1).

### 2.1. The Expert Panel

The panel included multidisciplinary specialists with extensive experience in the field. The team comprised two intensive care consultants, Prof. Tim Felton and Prof. Ignacio Martin-Loeches, a consultant in infectious diseases and medical microbiology, Dr. Anjaneya Bapat, and a clinical pharmacist specialising in critical care, Ms. Sarah Khorshid. Their diverse expertise ensured a comprehensive and balanced approach to developing the 5-step strategy. The small panel size enabled focused discussions, with members selected for their extensive clinical expertise, academic contributions, and leadership in critical care and infectious diseases.

### 2.2. Collaborative Development Process

The 5-step strategy was refined through an iterative and collaborative process involving multiple meetings and expert discussions. An initial alignment meeting was held on 6 November 2024, during which the panelists outlined the key challenges in IC diagnosis and management. This was followed by an advisory board meeting on 26 November 2024, where the team further analysed barriers to effective diagnosis and finalised the five-step strategy. Subsequent refinements were made through email correspondence to ensure all expert perspectives were considered. The five steps were developed with a focus on clinical feasibility. They incorporate tools and techniques that are either already in use or readily implementable in most ICUs in the UK and Ireland. It was also designed to be adaptable and scalable, allowing for future input from additional stakeholders and validation across diverse ICU settings [11].

The strategy was developed with a focus on adult ICU patients. While the principles of the strategy may be broadly applicable, its implementation in paediatric populations would require further validation and adaptation to account for differences in risk factors, diagnostic challenges, and antifungal pharmacokinetics.

### 2.3. Intended Clinical Impact

The 5-step strategy serves as a practical tool for clinicians managing IC in ICUs. By clarifying best practices and enhancing diagnostic efficiency, the pragmatic strategy aims to accelerate early diagnosis, reduce variability in care, and ultimately improve patient outcomes. It also promotes antifungal stewardship, ensuring more judicious use of antifungal therapy. While the strategy was specifically designed for ICUs in the UK and Ireland, its principles are broadly applicable to ICUs worldwide. However, its implementation may require local adaptation to account for variations in resource availability, healthcare infrastructure, and regional epidemiological factors [11]. Cost analysis was not the primary focus of this manuscript; however, the financial implications of implementing the 5-step strategy are an important consideration. Future studies should explore the cost-effectiveness of this approach to ensure its feasibility and sustainability across diverse healthcare settings.

### 2.4. Evidence-Based Strategy

The suggested strategy aligns with the 2025 global guidelines for the diagnosis and management of candidiasis, developed by the European Confederation for Medical Mycology (ECMM) in collaboration with the International Society for Human and Animal Mycology (ISHAM) and the American Society for Microbiology (ASM) through a rigorous, multidisciplinary process to ensure global applicability and evidence-based recommendations [11]. Blood cultures (two or three sets) remain the cornerstone of diagnosis and are strongly recommended, despite their limited sensitivity, aligning with Step 2 (Baseline Tests). The ECMM guidelines strongly recommend the use of biomarkers, such as BDG and mannan antigen/anti-mannan antibody assays, for diagnosing candidaemia or IC, but only in conjunction with clinical parameters, other biomarkers, or diagnostic tools [11]. Biomarkers should not be used as standalone diagnostic methods due to the potential for false positives or negatives [11]. Instead, they are most effective when integrated into a broader diagnostic framework, such as the 5-step strategy [11]. BDG testing aligns with Step 3 (Perform BDG Testing), where results are interpreted alongside clinical suspicion and imaging findings [11]. Direct microscopy and histopathology are also strongly recommended for tissue samples to confirm IC, particularly in cases of suspected deep-seated infections, corresponding to Step 4a (Histology and Biopsy Testing) [11]. Molecular techniques, including Candida PCR, are moderately supported for blood samples, particularly when rapid identification is needed, and MALDI-TOF MS is strongly recommended for species identification from culture isolates [11]. These methods align with Step 4c (MALDI-TOF MS) for rapid identification and targeted management. Imaging studies, such as CT or MRI, are strongly recommended for suspected deep-seated infections, corresponding to Step 4b (Imaging & Risk Scores), where imaging guides biopsy site selection and confirms organ involvement [11].

The 5-step strategy also aligns with the 2019 guidelines of the ESICM/ESCMID (European Society of Intensive Care Medicine/ European Society of Clinical Microbiology and Infectious Diseases) [15] and the 2024 FUNDICU (Fungal Infections in the ICU) consensus on managing invasive fungal diseases in the ICU [16].

### 2.5. Early Diagnosis and Rule-Out Tools

Effective management of IC in ICUs demands the rational use of antifungal therapies (AFT) [1,17]. A targeted ICU strategy reduces the likelihood of under-treatment in genuinely at-risk patients while preventing the overuse of antifungal agents in those without significant risk factors. Over-prescription, whether as empiric or prophylactic antifungal use, presents numerous challenges. It fosters the emergence of resistant *Candida* spp., such as *Candidozyma auris* (formerly *Candida auris*) and multidrug-resistant *N. glabrata*, which complicate future treatment efforts. Additionally, inappropriate antifungal use contributes to inefficiencies in treatment, increased healthcare costs, and unnecessary adverse effects, including drug toxicity and interactions [18]. These challenges underscore the importance of early diagnosis and tailored interventions to improve patient outcomes.

IC predominantly affects high-risk ICU patients, with a range of contributing factors. These include prolonged ICU stays, the use of central venous catheters, broad-spectrum antibiotics, sepsis, surgery, and total parenteral nutrition [5,19,20]. Colonisation with *Candida* spp., especially when coupled with other risk factors, serves as a strong predictor of IC [5]. Furthermore, underlying conditions such as immunosuppression, diabetes, malignancies, dialysis, and chemotherapy further elevate the risk of developing IC [18].

Risk assessments play a complementary role alongside diagnostic tests in identifying ICU patients at high risk. These assessments are crucial for justifying the initiation of empirical antifungal therapy. To improve predictive accuracy, validated risk prediction scores that incorporate specific patient demographic and clinical factors are recommended [18]. Several predictive tools have been developed to enhance early diagnosis and guide antifungal use in ICUs (Table 1) [18,21]. One notable example is the Candida Score, which identifies ICU patients with hospital-acquired severe sepsis or septic shock who may benefit from early antifungal treatment. A score greater than three indicates a high likelihood of IC, while a score of three or lower suggests a low probability [22]. Despite their inclusion in local hospital guidelines, the implementation of these tools in routine clinical practice remains inconsistent.

### 2.6. Diagnostic Challenges and Resource Disparities in Identifying IC in UK and Ireland ICUs

The expert panel identified numerous challenges in diagnosing IC within ICUs across the UK and Ireland. Despite blood cultures being the gold standard, their utility is significantly limited by prolonged time to positivity, often up to five days, and low sensitivity, particularly in detecting deep-seated infections such as intra-abdominal candidiasis, where the yield is approximately 50% [2,3,29,30]. However, evidence suggests that combining blood cultures with advanced diagnostic tools, such as molecular assays, can improve sensitivity and reduce diagnostic delays [11,31]. MALDI-TOF MS is available in many hospitals and offers rapid, accurate identification of major *Candida* spp. from cultured growth, but it cannot directly identify organisms from positive blood cultures without prior sub-culturing [2,3,29]. While its effectiveness depends on proper sample preparation, MALDI-TOF MS databases for *Candida* are well-developed, unlike those for broader fungi, which face limitations in standardisation and database representation [12]. Despite these challenges, MALDI-TOF MS is increasingly being adopted into routine diagnostic workflows [13]. However, barriers to its adoption include the need for specialised training, high initial costs, and workflow integration challenges, particularly in laboratories that also process mycobacterial blood cultures, as these workflows may conflict. Histological examination remains an important complementary diagnostic method, particularly for suspected deep-seated infections [32]. Tissue biopsies analysed with specialised fungal stains, such as periodic acid–Schiff (PAS) or Grocott’s methenamine silver (GMS), provide definitive evidence of tissue invasion [32]. However, the invasive nature of biopsies and delays in processing samples restrict their use to situations where they can be effectively combined with other diagnostic methods [32]. Non-culture-based tests (NCBTs), such as BDG assays, mannan/anti-mannan serological tests, T2 magnetic resonance Candida assay (T2Candida), and molecular diagnostics like multiplex Candida real-time PCR, are increasingly used for early diagnosis [2,3,11,29]. These biomarkers, while promising, face limitations due to variability in sensitivity, specificity, and overall clinical utility [30]. Such inconsistencies highlight the need for clearer guidelines and improved clinician education to maximise their potential. While BDG testing offers a valuable non-culture-based diagnostic option and can be a pragmatic approach, as shown in Figure 1, its high rate of false positives limits its reliability as a primary decision point in diagnostic algorithms [11]. This limitation may lead to unnecessary antifungal use, increasing the risk of resistance and treatment-related complications. Additionally, an antifungal stop strategy based solely on a negative BDG result does not account for false negatives, which can occur in cases of tissue-localised infections or when antifungal therapy suppresses BDG production [33]. The expert panel agreed on the importance of integrating BDG results with clinical judgment, imaging, and other diagnostic tools to avoid premature discontinuation of therapy, which is supported by the latest ECMM guidelines [11].

Resource disparities between hospitals also pose significant barriers to timely diagnosis. Some hospitals, e.g., teaching hospitals, with access to in-house BDG testing, may offer turnaround times of 24–48 h. In contrast, other hospitals rely on external laboratories, leading to delays of 7–14 days [34]. Additionally, variability in diagnostic practices, shaped by differences in resource availability, clinician expertise, and protocol adherence, further complicates timely and accurate diagnosis [14]. Clinical challenges are exacerbated by overlapping symptoms and non-specific signs of IC, often resulting in misdiagnoses or inappropriate treatment strategies. The increasing prevalence of antifungal resistance, particularly multidrug-resistant strains, adds another layer of complexity to the management of IC [4,17,35,36,37]. Furthermore, the absence of rapid diagnostic tools and insufficient integration of antifungal stewardship into broader antimicrobial programmes contribute to the overuse and improper management of antifungal treatments.

Although predictive risk models and colonisation indices hold promise for earlier diagnosis, their inconsistent application limits their utility. This issue is compounded by inadequate clinician education on fungal disease diagnostics and associated risk factors, emphasising the need for widespread training and awareness initiatives [14]. Ultimately, the lack of standardisation in diagnostic protocols across the UK and Ireland underscores the need for uniform guidelines, equitable resource allocation, and the development of faster, more accessible diagnostic tools. Addressing these gaps is essential to improving outcomes for critically ill IC patients [14].

### 2.7. The Role of Antifungal Susceptibility Testing

Timely antifungal susceptibility testing is crucial in refining therapy by enabling clinicians to select the most effective drug for a given fungal infection [38]. Rapid access to these results ensures treatments are both targeted and effective [38]. In-house MALDI-TOF MS-based techniques are well established and offer quick turnaround times. However, routine implementation in daily clinical practice is essential for maximising their utility [38].

### 2.8. De-Escalation and Early Stop Considerations

De-escalating or discontinuing antifungal therapy at the appropriate time is critical for minimising toxicity, preventing the development of drug resistance, and optimising the use of ICU resources [39]. Effective management requires close monitoring of patient progress through indicators such as infection markers, vital observations, and symptomatic recovery.

Key steps in this process include transferring stable patients to lower-acuity settings, reviewing follow-up test results such as blood cultures and BDG assays, and adhering to local guidelines for the duration of empirical antifungal courses, typically 5–7 days [39].

A summary of the criteria used to guide de-escalation and discontinuation of AFT is shown in Table 2. It is important to note that not all criteria listed need to be met simultaneously; clinical judgment should guide decisions based on the specific patient context.

Local protocols should incorporate a combination of clinical tools, biomarker data, and microbiological findings to guide antifungal management effectively. Collaboration between intensivists, infectious disease specialists, microbiologists, and pharmacists is essential to achieving optimal outcomes. Early interventions, such as source control measures, further enhance the effectiveness of antifungal treatments and support earlier discontinuation. By developing comprehensive strategies that prioritise timely diagnostics, empirical guidelines, and collaborative care, ICUs can strike a balance between effective treatment, resource optimisation, and patient safety.

### 2.9. Patient Case 1

This patient case highlights how a negative BDG assay, supported by clinical stability and negative microbiological tests, may allow safe discontinuation of antifungal therapy, promoting antifungal stewardship while avoiding unnecessary treatment: 

A 58-year-old man was admitted to the ICU after an emergency colectomy for bowel perforation. Despite broad-spectrum antibiotics, he developed persistent fever, hypotension requiring vasopressors, and rising inflammatory markers, prompting suspicion of IC. His risk factors included recent abdominal surgery, prolonged ICU stay, central venous catheter use, and total parenteral nutrition. Empirical antifungal therapy with anidulafungin was initiated, and a BDG assay was sent to assess for fungal infection. The BDG result was negative (<60 pg/mL), strongly suggesting the absence of IC. Blood cultures and fungal PCR were also negative. Based on these findings, antifungal therapy was discontinued after three days. Following the cessation of antifungals, the patient’s fever resolved, and he remained stable with improving inflammatory markers. By ICU day 10, he was weaned off vasopressors and extubated, later transitioning to the surgical ward for recovery.

### 2.10. Patient Case 2

The following patient case demonstrates how in-house susceptibility testing may ensure rapid confirmation of antifungal efficacy, allowing timely adjustments to therapy, reducing resistance risks, and optimising patient outcomes in high-risk ICU cases:

A 72-year-old woman was admitted to the ICU with septic shock secondary to peritonitis after a perforated appendicitis. Post-surgery, she remained febrile and hypotensive despite broad-spectrum antibiotics. Blood cultures grew *N. glabratus*, and antifungal therapy with micafungin was initiated. Given the rising concern of antifungal resistance in *Candida* spp., in-house antifungal susceptibility testing was promptly performed. Within 48 h, the susceptibility results confirmed the isolate’s resistance to fluconazole but susceptibility to micafungin. This rapid turnaround allowed the care team to confirm the appropriateness of the echinocandin therapy and avoid the use of ineffective antifungals. Over the next few days, the patient showed marked clinical improvement, with resolution of fever and haemodynamic stability. She was weaned off vasopressors and extubated by ICU day 9. Antifungal therapy with micafungin was continued for 14 days based on guidelines and clinical progress.

### 2.11. Diagnostic Innovations and Future Directions

The field of mycology is undergoing transformative advancements in diagnostic technologies designed to provide rapid, accurate, and reliable identification of fungal infections [40,41]. These innovations are crucial for the timely and effective management of patients, particularly those in critical care settings [40]. However, the widespread adoption of these tools is often hindered by high costs, making accessibility a significant challenge, especially in resource-limited settings where their impact could be most profound [40]. MALDI-TOF MS remains a cornerstone of fungal diagnostics, offering a substantial reduction in the time required to identify pathogens after cultures are obtained [42,43,44,45,46]. Efforts to expand fungal reference databases have significantly improved their accuracy, reaching 100% for identifying challenging species such as *C. auris* [47]. Additionally, advancements in molecular techniques, such as multi-single-nucleotide polymorphism (SNP) detection panels, complement MALDI-TOF MS by enabling the identification of drug-resistant phenotypes and supporting epidemiological studies, particularly in tracking resistance trends in *C. tropicalis* [48]. Despite its advancements, the technology remains predominantly limited to species-level identification post-culture, highlighting the need for broader application and integration into routine workflows.

Another innovation, lateral flow immunoassays (LFIAs), offers a rapid diagnostic option, particularly valuable in resource-limited environments [49]. By detecting IgG antibodies using recombinant enolase from *C. albicans*, LFIAs deliver results within 15 min [49]. While their specificity is high, the expert panel agrees that their moderate sensitivity makes them best suited as part of a multimodal diagnostic approach, complementing other methods for detecting IC [49].

Point-of-care (POC) diagnostics have emerged as a critical solution to the delays associated with traditional blood culture and serology methods [50,51]. Advanced technologies such as CRISPR-based platforms, loop-mediated isothermal amplification (LAMP), and microfluidic devices allow for faster, decentralised testing [50]. These tools minimise the time to diagnosis, reduce mortality rates, and lower healthcare costs. Notably, LAMP’s ability to function without thermal cycling makes it an especially promising approach when combined with compact microfluidic platforms for detecting *Candida* spp. [50]. Metagenomic next-generation sequencing (mNGS) represents another advancement in fungal diagnostics, particularly in cases where conventional methods fail [52]. By analysing genetic sequences, mNGS can identify rare, unknown, or mixed pathogens, making it indispensable for culture-negative scenarios [52]. Although it offers high sensitivity and specificity, its widespread use is restricted by high costs, complex fungal DNA extraction processes, and the need for a clinical context when interpreting results [52]. Advances in standardisation, cost reduction, and processing efficiency could enable mNGS to become a routine diagnostic tool in the future [52]. Artificial intelligence (AI) also holds great promise in fungal diagnostics, with models like InceptionV3 demonstrating exceptional accuracy in identifying *Candida* spp., including multidrug-resistant strains [53]. By streamlining workflows and providing cost-effective alternatives, AI has the potential to transform diagnostic processes, despite current limitations in methodology consistency and strain-specific identification [53]. Integrating biomarkers such as BDG with molecular markers and volatile organic compound (VOC) detection introduces non-invasive diagnostic pathways for fungal infections [54]. In particular, VOC breath analysis offers a painless and rapid method for diagnosing IC in critically ill patients. Diagnostics that target host immune responses, such as cytokine profiling, further enhance the ability to detect occult fungal infections [54]. Whole-genome sequencing (WGS) has proven invaluable for detecting resistance genes and tracking fungal outbreaks, providing critical insights into pathogen dynamics at the epidemiological level [55]. Nanotechnology is also driving progress with ultra-sensitive biosensors for detecting fungal antigens, while ImmunoPET imaging offers an innovative approach to visually localise invasive fungal infections using PET scans and fungal-specific antibodies [56]. Together, these advancements emphasise the need for ongoing research, validation, and investment to make cutting-edge diagnostics widely accessible. As these tools become integrated into clinical practice, they can transform the management of IC in critically ill patients, reducing mortality and improving outcomes on a global scale.

## 3. Conclusions

The diagnosis and management of IC in ICUs across the UK and Ireland remain significant challenges due to delays in diagnostic processes, resource disparities, and rising antifungal resistance. While advancements such as MALDI-TOF MS, lateral flow immunoassays, mNGS, and POC technologies offer promising solutions, their adoption is hindered by high costs, variability in application, and insufficient standardisation. Antifungal stewardship programmes and multidisciplinary collaboration are essential to ensure timely, effective, and targeted therapy, reducing unnecessary treatments and associated resistance risks.

The five-step IC diagnostic strategy offers a structured approach to help address these issues. By incorporating risk assessments and biomarkers, it improves diagnostic accuracy, supports timely and precise treatments, and promotes antifungal stewardship through standardised practices. By reducing disparities and enhancing consistency in IC management, the structured approach may help improve outcomes and reduce mortality in critically ill patients. This collaborative and practical strategy provides valuable support for effective IC management in ICU settings.

## Figures and Tables

**Table 1:** IC risk assessment prediction tools.

Prediction Score/Rule	Criteria	Comments/Utility	Reference(s)
Dupont Score	Total parenteral nutrition Broad-spectrum antibiotics *Candida* colonisation at ≥2 sites	Focuses on colonisation and clinical factors to identify risk.	Dupont et al. (2003) [23]
Candida Score	Multisite colonisation Severe sepsis Total parenteral nutrition Surgery Score ≥ 3.0 indicates risk	Simple, widely used score; validated for ICU patients.	Leon et al. (2006), Leroy et al. (2011) [22,24]
Ostrosky Rule	Prolonged ICU stay Broad-spectrum antibiotics *Candida* colonisation Central venous catheter Dialysis	Effective for early initiation of AFT.	Ostrosky-Zeichner et al. (2007) [25]
Nebraska Medical Centre Rule	Fever unresponsive to antibiotics Risk factors: total parenteral nutrition, broad-spectrum antibiotics, surgery, central lines	Used primarily for empiric antifungal therapy decisions.	Hermsen et al. (2011) [26]
Candidemia Rule	Prior broad-spectrum antibiotics Central venous catheter Total parenteral nutrition ICU stay >72 h ≥2 factors = risk	High sensitivity for detecting candidemia; focuses on commonly recognised ICU risk factors.	Guillamet et al. (2015) [27]
Other Notable Scores	Pitt Score for candidemia prognosis Emerging biomarkers (e.g., BDG, PCR, PNA-FISH) combined with scores	Biomarkers may enhance predictive accuracy when combined with clinical scores.	Vaquero-Herrero et al. (2017) [28]

AFT, antifungal Therapy; BDG, (1,3)-β-D-glucan; IC, invasive candidiasis; ICU, intensive care unit; PCR, polymerase chain reaction; PNA-FISH, peptide-nucleic acid fluorescent in situ hybridisation.

**Table 2:** Guide criteria for AFT de-escalation and early discontinuation.

**De-Escalation Criteria:**	
Confirmed *Candida* species and susceptibility	e.g., switching from echinocandins to fluconazole for susceptible *Candida albicans*
Clinical improvements	Indicators such as resolution of fever, stabilisation of blood pressure, and recovery of organ function.
Clearance of candidemia	Serial negative blood cultures confirm eradication of the infection.
**Early Discontinuation Criteria:**	
Low pre-test probability of IC	Based on clinical scoring systems like the Candida Score or Ostrosky rule.
Negative fungal biomarkers	Persistent negative BDG results and other diagnostic tests.
Effective source control	Removal of infected devices such as central lines or drainage of abscesses.
Role of biomarkers and molecular diagnostics	Biomarkers such as serial negative BDGs, along with molecular tests like PCR, play a crucial role in ruling out candidiasis. Negative results provide confidence in ceasing antifungal therapy, particularly in critically ill patients on broad-spectrum antifungals.

BDG, (1,3)-β-D-glucan; IC, invasive candidiasis; PCR, polymerase chain reaction.

## Data Availability

All data generated or analysed during this study are included in this published article.
